# Characteristics Associated with Being a High Consumer of Sweet Foods and Sugar-Sweetened Beverages among US Adults during the COVID-19 Pandemic, 2021

**DOI:** 10.3390/nu15102363

**Published:** 2023-05-18

**Authors:** Sohyun Park, Seung Hee Lee, Heidi M. Blanck

**Affiliations:** 1 Division of Nutrition, Physical Activity, and Obesity (DNPAO), National Center for Chronic Disease Prevention and Health Promotion (NCCDPHP), Centers for Disease Control and Prevention (CDC), 4770 Buford Highway, NE, Atlanta, GA 30341, USA; xde5@cdc.gov (S.H.L.); hcb3@cdc.gov (H.M.B.); 2 Division of Overdose Prevention, National Center for Injury Prevention and Control, Centers for Disease Control and Prevention, 4770 Buford Highway, NE, Atlanta, GA 30341, USA

**Keywords:** added sugars, sweet foods, desserts, sugar-sweetened beverage, COVID-19 pandemic, US adults, diet

## Abstract

Background: The COVID-19 pandemic impacted some dietary habits of Americans. Objective: We examined characteristics associated with a high intake of sweet foods and sugar-sweetened beverages (SSB) during the COVID-19 pandemic among US adults. Design: This was a cross-sectional study. Participants/settings: The SummerStyles survey data were collected in 2021 among 4034 US adults (≥18 years). Main outcome measures: The frequencies were measured of consuming various sweet foods (chocolate/candy, doughnuts/sweet rolls/Danish/muffins/Pop-Tarts, cookies/cake/pie/brownies, and ice cream/frozen desserts) and SSB (regular sodas, sweetened coffee/tea drinks fruit drinks, sports drinks, and energy drinks) during the COVID-19 pandemic. The responses were categorized into 0, >0 to <1, 1 to <2, and ≥2 times/day. The descriptive variables were sociodemographics, food insecurity, weight status, metropolitan status, census regions, and eating habit changes during the COVID-19 pandemic. Statistical analyses performed: Multinomial regressions were used to estimate adjusted odds ratios (AOR) for being a high consumer of sweet foods and SSB after controlling for characteristics. Results: During 2021, 15% of adults reported consuming sweet foods ≥2 times/day, and 30% reported drinking SSB ≥2 times/day. The factors that were significantly associated with greater odds of high sweet food intake (≥2 times/day) were lower household income (AOR = 1.53 for <$35,000 vs. ≥$100,000), often/sometimes experiencing food insecurity (AOR = 1.41 vs. never), and eating more sweet foods than usual since start of the pandemic (AOR = 2.47 vs. same as usual). The factors that were significantly associated with greater odds of high SSB intake (≥2 times/day) were males (AOR = 1.51), lower education (AOR = 1.98 for ≤high school; AOR = 1.33 for some college vs. college graduate), currently having children (AOR = 1.65), living in nonmetropolitan areas (AOR = 1.34), and drinking more SSB than usual since the pandemic began (AOR = 2.23 vs. same as usual). Younger age, Black race, and reductions in consumption during COVID-19 were related to lower sweet food and SSB intakes. Conclusions: Our findings, which identified high consumers of sweet foods or SSB, can be used to inform efforts to reduce consumers’ added sugars intake during pandemic recovery and support their health.

## 1. Introduction

Added sugars intake is high among US adults. In 2017–2018, the mean added sugars intake among US adults aged ≥20 years was 17.1 teaspoons (tsp)/day, 19.2 tsp/day for men and 15.1 tsp/day for women, based on the National Health and Nutrition Examination Survey (NHANES) [1]. These values were much higher than the American Heart Association’s recommendation to consume less than 9 tsp/day for men and 6 tsp/day for women [2]. The high intake of added sugars is a public health concern because a greater intake of added sugars has been associated with adverse health risks in adults, including a higher risk of cardiovascular disease mortality and metabolic disease [3,4]. Added sugars come from sugary foods (e.g., sweet bakery products, candy, and other desserts) and sugar-sweetened beverages (SSB), with SSB being the leading source of added sugars among US adults [5]. As a result of the adverse health consequences related to added sugars intake, the 2020–2025 Dietary Guidelines for Americans (DGA) recommend that Americans aged ≥2 years should consume less than 10% of their total daily calories from added sugars [6]. Furthermore, supporting healthful eating habits by reducing the added sugars intake of individuals aged 2 years and over is a Healthy People 2030 priority objective of the US Department of Health and Human Services [7].

Previous studies reported that eating habits of US adults may have changed during the COVID-19 pandemic [8,9,10,11], and that this could be partially been due to stress, anxiety, and disruption to daily lives related to the COVID-19 pandemic [12,13,14,15]. For instance, a study conducted among adults living in L.A. County in 2020 (*n* = 1070) reported that 28% of adults were eating healthier food and 25% were eating less healthy food compared to their diet before the COVID-19 pandemic; 47% reported not changing their diet [11]. Of these studies that examined changes in eating habits, only a few studies (with small sample sizes) focused on sweet foods and/or SSB in the early phase of the COVID-19 pandemic [9,10]. The exception was one larger study of US adults (*n* = 3916) in 2020 [8]. For example, Sadler and colleagues reported that 41% of US adults had increased their intake of sweets/desserts during the COVID-19 pandemic in 2020 (*n* = 428) [9]. In our previous study conducted in June 2020, we found that 16% of US adults reported often/always eating more unhealthy snacks and desserts and 10% reported often/always drinking more SSB during 3 months of the COVID-19 pandemic (*n* = 3916) [8]. However, in the previous study, we did not quantify the dietary intakes of sweet foods and SSB. Therefore, in this present study, we explored various factors associated with the high consumption of sweet foods and SSB—among US adults, using the 2021 SummerStyles survey data—during the middle phase of the COVID-19 pandemic.

## 2. Methods

### 2.1. Study Sample and Survey Administration

We conducted a cross-sectional study using the SummerStyles survey data collected online in 2021 by Porter Novelli Public Services [16]. SummerStyles survey data are collected through Ipsos’ KnowledgePanel^®^, an online panel representative of the noninstitutionalized US population. Using a probability-based sampling method by address, panel members are randomly recruited by mail. Internet access and/or a laptop or tablet were provided if households needed them. The panel is continuously refilled, and keeps about 60,000 panelists. The survey asks various questions on health-related knowledge, behaviors, and attitudes around important public health topics and health conditions. This analysis was exempt from the Centers for Disease Control and Prevention (CDC) Institutional Review Board, because personal identifiers were absent in the data provided to the CDC.

**Figure 1:**
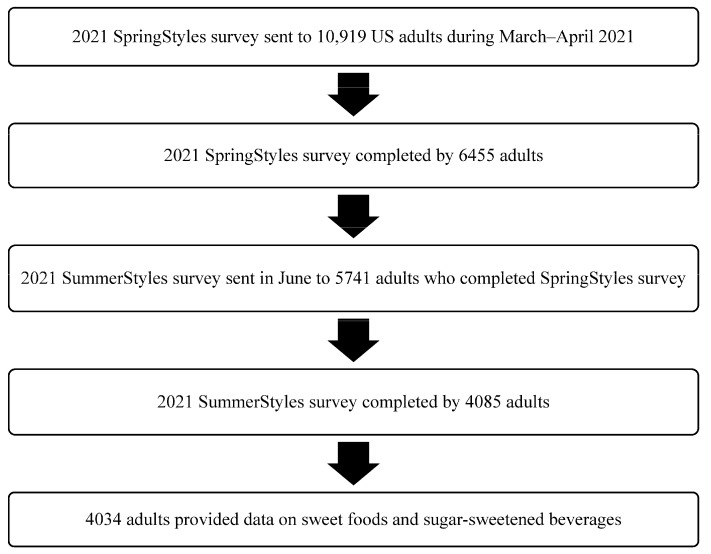
Analytic sample flow chart for SummerStyles survey among US adults, 2021.

As described in Figure 1, the SummerStyles survey was sent to persons who participated in the SpringStyles survey (an initial wave). The survey respondents received cash-equivalent reward points for their participation (worth approximately $10). From March to April 2021, the SpringStyles survey was sent to 10,919 panelists (≥18 years), and 6455 completed the survey, a response rate of 59.1%. During June 2021, the SummerStyles survey was sent to 5741 adults who completed the SpringStyles survey. The SummerStyles survey was completed by 4085 adults (≥18 years), a response rate of 71.2%. The data were weighted to match the 2019 Census’ American Community Survey proportions using 9 factors: age, sex, race/ethnicity, education, household income, household size, census region, metropolitan status, and parental status of children aged 12–17 years old. Of 4085 adults who completed the 2021 SummerStyles Survey, 51 adults (1.2%) with missing data on outcome variables (i.e., intake of sweet foods or SSB) were excluded from our study, leaving a final analytic sample of 4034 adults.

### 2.2. Outcome Variables

The outcome variables of interest that were reported were the frequencies of consuming sweet foods and SSB. We created a composite variable for sweet foods using the following 4 questions. During the past month how often did you eat: (1) Chocolate, or any other types of candy? Do not include sugar-free candy; (2) Doughnuts, sweet rolls, Danish, muffins, (pan dulce) or Pop-Tarts? Do not include sugar-free items; (3) Cookies, cake, pie, or brownies? Do not include sugar-free kinds; and (4) Ice cream or other frozen desserts? Do not include sugar-free kinds. A composite variable for SSB was created using the following 5 questions. During the past month, how often did you drink: (1) Regular soda or pop that contains sugar? Do not include diet soda; (2) Coffee, including lattes and tea, including bottled tea, that was sweetened with sugar or honey? Do not include drinks with things like Stevia; (3) Sports drinks such as Gatorade or Powerade? Do not include diet drinks; (4) Energy drinks like Red Bull, Monster, NOS, 5-Hour Energy, or Full Throttle? Do not include diet drinks; and (5) Sweetened fruit drinks, such as Kool-Aid, cranberry, and lemonade? Do not include 100% fruit juice. For each question on sweet food and SSB intake, the response options were none, <1 time/week, 1–6 times/week, 1 time/day, 2 times/day, and ≥3 times/day. The weekly intake was converted to daily intake. For example, 1–6 times/week was converted to 0.5 times/day (3.5 divided by 7) and ≥3 times/day was converted to 3 times/day. To estimate the frequency of total daily sweet food intake, we added the responses from the intake of chocolate/candy, doughnuts/sweet rolls/Danish/muffins/Pop-Tarts, cookies/cake/pie/brownies, and ice cream/frozen desserts. For total the SSB intake, we added the responses from the intake of regular sodas, sweetened coffee/tea drinks, fruit drinks (excluding 100% juice), sports drinks, and energy drinks. Four mutually exclusive categories (0, >0 to <1, 1 to <2, and ≥2 times/day) were created for sweet food intake and SSB intake. We defined high consumers as those consuming sweet foods or SSB ≥2 times/day.

### 2.3. Descriptive Variables

The descriptive variables included sociodemographic characteristics, food insecurity, weight status, metropolitan status, census regions, and reported eating habit changes during the COVID-19 pandemic. The sociodemographic variables were age (18–24, 25–44, 45–64, or ≥65 years), sex (male, female), race/ethnicity (non-Hispanic [NH] Black, Hispanic, NH other/multiracial, or NH White), education level (≤high school, some college, or college graduate), marital status (married, not married), annual household income (<$35,000, $35,000–$74,999, $75,000–$99,999, or ≥$100,000), and currently having children aged <18 years (yes, no). Food insecurity was categorized as often/sometimes, seldom, never, or do not know/not sure based on the following question: “Thinking about the past year, during the COVID-19 pandemic, how often did you rely on only a few kinds of low-cost food to feed yourself or household members because there wasn’t enough money to buy food? Low-cost food can include items such as macaroni and cheese, peanut butter, pasta, ramen, instant soups, and sugary drinks (lacking variety with little or no protein, vegetables, or fruit).” The weight status was classified into 3 categories based on the respondent’s self-reported height and weight, which were used to calculate body mass index (BMI): underweight/healthy weight (BMI < 25 kg/m^2^), overweight (BMI 25 to <30 kg/m^2^), and obesity (BMI ≥ 30 kg/m^2^) [17]. Underweight and healthy weight were combined into a single category because only 2% of the adults were underweight. The metropolitan status was nonmetropolitan or metropolitan. The census regions of residence were Northeast, Midwest, South, or West [18]. Any reported changes in eating habits were assessed using the following questions: “Has your consumption of the following food items changed since the start of the COVID-19 pandemic?” Two items were evaluated from the following question: (1) “Desserts and sweets like cookies, cakes, ice cream, doughnuts, and candy” and (2) “Sugary drinks like regular soda, fruit drinks, sports or energy drinks, sweetened coffee/teas drinks.” The response options were as follows: much less than usual, a little less than usual, the same as usual, a little more than usual, and much more than usual. For our study, we categorized the responses into less than usual, same as usual, or more than usual.

### 2.4. Statistical Analysis

For the unadjusted bivariate analysis, we used descriptive statistics to examine the factors associated with high sweet food or SSB intake during the past month using χ^2^ tests. A *p* ≤ 0.05 was used to define statistical significance. We used two multinomial logistic regression models for the adjusted analysis to calculate adjusted odds ratios (AORs) and 95% confidence intervals (CIs) for factors associated with high sweet food and SSB intake. Each model included all variables in one model, and the reference group was consuming <1 time/day. Of those 4034 adults with outcome data, the sample size decreased to 3937 adults for the sweet food regression model and 3922 adults for the SSB regression model because of missing data on some explanatory variables. All statistical analyses were performed with the Statistical Analysis Software (SAS) Version 9.4 (SAS Institute Inc., Cary, NC, USA), using survey procedures to account for the sampling weights.

## 3. Results

Overall, 35% of the participants were aged 25–44 years old; 51% were female, 63% were NH White adults, 38% had a high school education or less, 57% were married adults, and 39% had annual household income of ≥$100,000. Most adults (72%) did not currently have children under 18; 59% never had food insecurity in the past year; 35% had obesity; 87% lived in metropolitan areas, and 38% resided in the South (Table 1).

In 2021 during the COVID-19 pandemic, 15% of the adults reported consuming sweet foods ≥2 times/day and were henceforth called high consumers. Based on bivariate analyses, high sweet food intake was significantly associated with age, race/ethnicity, education level, annual household income, food insecurity, metropolitan status, census regions of residence, and changes in sweet food intake since the COVID-19 pandemic began (χ^2^ tests, *p* < 0.05; Table 1). For instance, the proportion of high consumers (≥2 times/day) for sweet foods was greatest among adults who were older (≥65 years), of Hispanic or NH White groups, had ≤ high school education, had annual household income < $35,000, had food insecurity, lived in a nonmetropolitan area, resided in the Northeast, and reported eating more sweet foods since the COVID-19 pandemic began. Based on the multinominal logistic regression model using sweet food intake < 1 time/day as a reference group, that factors that were significantly associated with greater odds of high sweet food intake (≥2 times/day) were lower household income (AOR = 1.53 for <$35,000 vs. ≥$100,000), often/sometimes experiencing food insecurity (AOR = 1.41 vs. never), and eating more sweet foods than usual since the pandemic began (AOR = 2.47 vs. same as usual). The factors that were significantly associated with lower odds of high sweet food intake (≥2 times/day) were younger adults (AOR range: 0.42–0.64 vs. older ≥ 65 years), NH Black adults (AOR = 0.62 vs. NH White adults), and eating fewer sweet foods than usual since the pandemic began (AOR = 0.46 vs. same as usual) (Table 1). In a sensitivity analysis, when the variable on changes in sweet food intake since the start of the COVID-19 pandemic was removed from the model, the findings remained similar, except that household income became nonsignificantly associated with sweet food intake ≥2 times/day (Supplemental Table S1).

During the COVID-19 pandemic, 30% of adults reported drinking SSB ≥2 times/day in 2021. Based on bivariate analyses, a high SSB intake was significantly associated with age, sex, race/ethnicity, education level, annual household income, currently have children <18 years, food insecurity, metropolitan status, census regions of residence, and changes in SSB intake since the COVID-19 pandemic began (χ^2^ tests, *p* < 0.05; Table 2). For instance, the proportion of high consumers (≥2 times/day) for SSB was greatest among adults aged 25–44 years or 45–64 years, males, Hispanic or NH White adults, those with ≤high school education, those with an annual household income < $35,000, adults who currently have children, people with food insecurity or those who were not sure, adults living in nonmetropolitan areas, adults residing in the Northeast, and people who reported drinking more SSB than usual since the COVID-19 pandemic began. Based on the multinominal logistic regression model using SSB intake <1 time/day as a reference group, the factors that were significantly associated with greater odds of high SSB intake (≥2 times/day) were males (AOR = 1.51), lower education (AOR = 1.98 for ≤high school; AOR = 1.33 for some college vs. college graduate), currently having children (AOR = 1.65), living in nonmetropolitan areas (AOR = 1.34), and drinking more SSB than usual since the pandemic began (AOR = 2.23 vs. same as usual). The factors that were significantly associated with lower odds of high SSB intake (≥2 times/day) were young people aged 18–24 years (AOR = 0.47 vs. older people ≥ 65 years), NH Black adults (AOR = 0.64 vs. NH White adults), and drinking less SSB than usual since the pandemic began (AOR = 0.60 vs. same as usual). In a sensitivity analysis, when the variable on changes in SSB intake since the start of the COVID-19 pandemic was removed from the model, the findings remained similar, except that food insecurity became significantly associated with SSB intake ≥2 times/day (AOR = 1.34, 95% CI = 1.03–1.74; Supplemental Table S1).

## 4. Discussion

In the present study, 14.9% of adults reported eating sweet foods ≥2 times/day, and 29.9% reported drinking SSB ≥2 times/day during the COVID-19 pandemic in 2021. Although our findings cannot be directly compared to those of the NHANES, based on 2015–2018 NHANES data, about 30% of US adults were classified as high added sugars consumers (i.e., consuming > 15% of total energy from added sugars), and the top sources of added sugars were SSB and sweet bakery products among high consumers [19]. Although we were unable to find any published peer-reviewed studies reporting solely high sweet food intake (without beverage sources) among US adults, a US Department of Agriculture (USDA) study reported that 61% of US adults aged ≥20 years consumed any sweet foods (without beverage sources) on a given day using the 2015–2018 NHANES data [20]. Additionally, the prevalence of high SSB intake (≥2 times/day) was lower in our study than in a previous study that found 37.8% of US adults reported drinking SSB ≥2 times/day in 2014, using the same questions in the same survey [21].

Both sweet foods and SSB contribute to total added sugars intake. In the current study, (1) the odds of high sweet food intake (≥2 times/day) were significantly lower among younger adults (vs. older adults) and NH Black adults (vs. NH White adults), and (2) the odds were significantly greater among lower income adults (vs. higher household income) and those with food insecurity (vs. those without). These findings were somewhat consistent with the previous USDA report that used similar classifications for sweet foods and SSB, which found that the prevalence of any sweet food (excluding SSB) intake was lower in young adults than older adults (55% vs. 70%, respectively), and lower in NH Black than NH White adults (56% vs. 64%, respectively) on a given day in 2015–2018 [20]. However, inconsistently with our findings, the USDA report showed that the prevalence of sweet food intake was significantly greater among higher-income adults than lower-income adults (64% vs. 53%, respectively) [20]. Somewhat inconsistently with our findings, another study conducted in June 2020, which was an earlier phase of the COVID-19 pandemic, found that younger adults, NH Black adults, lower-income people, and adults with obesity reported consuming more unhealthy snacks and desserts during 3 months of the COVID-19 pandemic [8]. It is possible that the eating habits of unhealthy foods may have shifted over time during the pandemic among US adults, due to potential changes in stress, mobility, work roles, and caretaking. Moreover, our findings of an association between food insecurity and high sweet food intake provide important information related to the ongoing need to support healthful foods reaching those most in need during public health emergencies. Our findings are consistent with prior studies showing that food insecurity is inversely associated with diet quality, which can impact short- and long-term health [22].

In 2021 during the COVID-19 pandemic, the odds of high SSB (≥2 times/day) were significantly lower among younger adults and NH Black adults, while the odds were significantly greater among men, adults with lower education, adults who had children, and those living in nonmetropolitan areas compared to their counterparts in our study. Interestingly, the prevalence of high SSB intake (≥2 times/day) among 18–24 year-olds was lower in that from our study conducted in 2021 during the COVID-19 pandemic compared to a previous study conducted in 2014 (22.2% vs. 36.5%, respectively) [21]. However, the prevalence of high SSB intake among older adults (≥65 years) was similar in our study (28.4%) and the 2014 study (28.2%) [21]. Unlike our findings, findings from the 2020 study (conducted in an earlier phase of the COVID-19 pandemic) reported that younger adults and NH Black adults had significantly greater odds for consuming more SSB [8], although this 2020 study did not measure the frequency of SSB intake. This shift in characteristics related to high SSB intake may be partially due to changes in work patterns (e.g., unemployment or loss of income during the pandemic), which might have resulted in economic deprivation and disparities in certain populations [23,24]. Food insecurity was significantly associated with consuming SSB 1 to <2 times/day in our study. The COVID-19 pandemic may have more profoundly impacted individuals with food insecurity; a previous study conducted in the San Francisco Bay area reported that the daily SSB intake was significantly higher among those who experienced financial hardship during the COVID-19 pandemic than those without hardship [23]. Additionally, persons’ access to SSB may have shifted during the COVID-19 pandemic (e.g., vending machines at work, retail closures, etc.). We also found that adults who have children reported high SSB intake during the COVID-19 pandemic. Similarly to our findings, another study showed that the prevalence of drinking SSB ≥1 time/day was significantly higher among US adults living in nonmetropolitan vs. metropolitan areas (30.9% vs. 24.8%) based on the 2017 Behavioral Risk Factor Surveillance System [25].

Our study found that compared to before the pandemic, 20.0% of adults reported consuming more sweet foods, and 10.6% of adults reported consuming more SSB. Supporting healthful eating habits by reducing the added sugars intake of individuals is a Healthy People 2030 priority [7], and this remains an opportunity as the nation moves into endemic and/or pandemic recovery—to advance messaging about the importance of healthy eating so that the body can protect itself against both chronic diseases and infectious disease severity [6,26].

This study has at least four limitations. Firstly, the causality cannot be determined, because the SummerStyles survey is a cross-sectional survey. Secondly, the data may have been subject to recall and/or social desirability bias, because the SummerStyles survey data are self-reported. Thirdly, the survey measured the frequencies of sweet food and SSB intake, and not the quantities. However, a study has shown moderate relative validity for snacks and beverages [27]. Fourthly, our findings may not be generalizable to the entire US adult population because the initial sample was chosen from the larger online panel that people were willing to be part of. However, the data were weighted to be similar to Current Population Survey proportions.

## 5. Conclusions

About 1 in 6 adults reported eating sweet foods ≥2 times/day, and 1 in 3 adults reported drinking SSB ≥2 times/day during the COVID-19 pandemic in 2021. The factors associated with a high intake of sweet foods or SSB varied among US adults during the COVID-19 pandemic; thus, tailored intervention strategies may be needed to reduce added sugars intake. Our findings that identified high consumers of sweet foods or SSB can be used to inform efforts such as the following: educational campaigns using the nutrition facts label, counseling by healthcare providers, and implementation of nutrition standards and food service guidelines in organizations—to reduce consumers’ added sugars intake during the pandemic recovery and to support their health.

## Figures and Tables

**Table 1:** Characteristics of respondents (US adults) and their associations with consuming sweet foods during COVID-19 pandemic, SummerStyles Survey, 2021.

	All Respondents	Sweet Food ^a^ Intake during the Past Month					
		Bivariate Analysis ^b^				Multinomial Analysis ^c^	
Characteristic	% ^d^	0 Times/Day % ^d^ ± SE	>0 to <1 Time/Day % ^d^± SE	1 to <2 Times/Day % ^d^ ± SE	≥2 Times/Day % ^d^ ± SE	1 to <2 Times/Day AOR (95% CI)	≥2 Times/Day AOR (95% CI)
Total (*n* = 4034) ^e^	100	5.6 ± 0.5	51.8 ± 0.9	27.6 ± 0.8	14.9 ± 0.7	—	—
Age							
18–24 years	10.6	**3.5 ± 1.5**	**54.4 ± 4.2**	**30.2 ± 3.9**	**11.9 ± 2.8**	0.71 (0.45, 1.12)	**0.42 (0.22, 0.79)**
25–44 years	34.8	**7.5 ± 1.0**	**51.6 ± 1.7**	**25.8 ± 1.4**	**15.1 ± 1.2**	**0.61 (0.47, 0.79)**	**0.64 (0.46, 0.88)**
45–64 years	33.5	**5.3 ± 0.7**	**54.6 ± 1.4**	**26.4 ± 1.2**	**13.8 ± 1.0**	**0.64 (0.52, 0.80)**	**0.59 (0.45, 0.78)**
≥65 years	21.1	**3.9 ± 0.6**	**46.7 ± 1.6**	**31.4 ± 1.5**	**18.0 ± 1.2**	Reference	Reference
Sex							
Male	48.6	6.5 ± 0.7	52.9 ± 1.3	26.4 ± 1.1	14.3 ± 0.9	0.89 (0.74, 1.07)	0.94 (0.75, 1.18)
Female	51.4	4.7 ± 0.6	50.9 ± 1.3	28.8 ± 1.2	15.6 ± 1.0	Reference	Reference
Race/ethnicity							
Black, non-Hispanic	11.7	**10.2 ± 2.1**	**56.4 ± 3.2**	**21.5 ± 2.5**	**12.0 ± 2.2**	**0.62 (0.45, 0.86)**	**0.62 (0.40, 0.97)**
Hispanic	16.3	**6.3 ± 1.3**	**53.0 ± 2.9**	**25.1 ± 2.6**	**15.6 ± 2.0**	0.85 (0.62, 1.15)	0.99 (0.68, 1.43)
Other/multiracial, non-Hispanic	8.8	**6.0 ± 1.6**	**54.2 ± 3.3**	**28.2 ± 2.9**	**11.5 ± 2.0**	0.89 (0.64, 1.23)	0.71 (0.45, 1.13)
White, non-Hispanic	63.2	**4.5 ± 0.5**	**50.4 ± 1.1**	**29.4 ± 1.0**	**15.8 ± 0.8**	Reference	Reference
Education level							
High school or less	38.4	**7.5 ± 1.0**	**51.0 ± 1.7**	**25.1 ± 1.5**	**16.3 ± 1.3**	0.87 (0.68, 1.11)	0.95 (0.71, 1.28)
Some college	30.0	**4.0 ± 0.6**	**53.0 ± 1.7**	**29.3 ± 1.5**	**13.8 ± 1.2**	0.99 (0.94, 1.24)	0.91 (0.68, 1.21)
College graduate	31.6	**4.7 ± 0.6**	**51.8 ± 1.4**	**29.2 ± 1.3**	**14.3 ± 0.9**	Reference	Reference
Marital status							
Married	57.1	5.0 ± 0.5	51.4 ± 1.1	29.0 ± 1.0	14.5 ± 0.8	Reference	Reference
Not married	42.9	6.3 ± 0.8	52.4 ± 1.6	25.8 ± 1.4	15.5 ± 1.2	0.95 (0.77, 1.17)	1.04 (0.81, 1.34)
Annual household income							
<$35,000	19.9	**9.4 ± 1.5**	**48.2 ± 2.4**	**23.0 ± 2.0**	**19.3 ± 2.0**	1.04 (0.75, 1.44)	**1.53 (1.05, 2.23)**
$35,000–$74,999	27.5	**5.5 ± 0.9**	**52.2 ± 1.8**	**27.7 ± 1.6**	**14.6 ± 1.3**	1.05 (0.82, 1.34)	1.02 (0.76, 1.39)
$75,000–$99,999	13.7	**4.0 ± 0.9**	**52.2 ± 2.4**	**31.1 ± 2.2**	**12.7 ± 1.5**	1.20 (0.92, 1.56)	0.99 (0.70, 1.41)
≥$100,000	38.9	**4.2 ± 0.5**	**53.3 ± 1.4**	**28.8 ± 1.3**	**13.7 ± 0.9**	Reference	Reference
Currently have children (<18 years) (*n* = 4032)							
Yes	27.6	4.8 ± 0.8	52.9 ± 1.7	28.5 ± 1.6	13.8 ± 1.3	1.07 (0.86, 1.34)	0.86 (0.64, 1.17)
No	72.4	5.9 ± 0.6	51.5 ± 1.1	27.3 ± 1.0	15.4 ± 0.8	Reference	Reference
Food insecurity ^f^ (*n* = 4026)							
Often/sometimes	21.9	**5.4 ± 1.0**	**49.1 ± 2.3**	**27.6 ± 2.0**	**18.0 ± 1.7**	1.27 (0.98, 1.66)	**1.41 (1.03, 1.94)**
Seldom	12.8	**4.2 ± 1.2**	**50.9 ± 2.7**	**28.7 ± 2.3**	**16.2 ± 1.9**	1.20 (0.92, 1.56)	1.32 (0.93, 1.88)
Never	58.8	**5.0 ± 0.5**	**53.3 ± 1.1**	**27.7 ± 1.0**	**14.0 ± 0.8**	Reference	Reference
Do not know or not sure	6.5	**13.8 ± 3.0**	**50.1 ± 4.4**	**25.4 ± 4.0**	**10.7 ± 2.6**	1.09 (0.68, 1.72)	0.79 (0.44, 1.43)
Weight status ^g^ (*n* = 3957)							
Underweight/healthy weight	33.1	5.7 ± 0.8	51.3 ± 1.7	28.5 ± 1.6	14.4 ± 1.2	Reference	Reference
Overweight	32.3	4.9 ± 0.8	51.8 ± 1.6	28.8 ± 1.4	14.6 ± 1.1	1.05 (0.85, 1.31)	0.98 (0.74, 1.32)
Obesity	34.6	6.1 ± 0.9	52.3 ± 1.6	25.8 ± 1.4	15.8 ± 1.2	0.93 (0.73, 1.18)	0.98 (0.74, 1.30)
Metropolitan status							
Nonmetropolitan	13.4	**5.7 ± 1.3**	**54.3 ± 2.5**	**21.1 ± 2.0**	**18.9 ± 2.0**	**0.68 (0.51, 0.90)**	1.10 (0.80, 1.52)
Metropolitan	86.6	**5.6 ± 0.5**	**51.5 ± 1.0**	**28.7 ± 0.9**	**14.3 ± 0.7**	Reference	Reference
Census regions of residence							
Northeast	17.3	**5.7 ± 1.1**	**49.7 ± 2.2**	**27.6 ± 1.9**	**17.0 ± 1.6**	1.06 (0.82, 1.37)	1.20 (0.87, 1.66)
Midwest	20.8	**4.4 ± 0.9**	**48.4 ± 2.0**	**32.4 ± 1.9**	**14.7 ± 1.3**	**1.33 (1.05, 1.68)**	1.07 (0.79, 1.46)
South	38.1	**7.0 ± 0.8**	**52.7 ± 1.6**	**25.4 ± 1.3**	**14.8 ± 1.2**	Reference	Reference
West	23.8	**4.2 ± 0.8**	**55.0 ± 2.0**	**27.0 ± 1.7**	**13.8 ± 1.3**	1.02 (0.80, 1.29)	1.02 (0.74, 1.39)
Changes in sweet food intake since start of the COVID-19 pandemic (*n* = 4020)							
Less than usual	18.8	**11.3 ± 1.5**	**63.6 ± 2.1**	**16.6 ± 1.6**	**8.6 ± 1.2**	**0.46 (0.35, 0.59)**	**0.46 (0.32, 0.65)**
Same as usual	61.1	**5.3 ± 0.6**	**52.4 ± 1.2**	**28.2 ± 1.1**	**14.2 ± 0.8**	Reference	Reference
More than usual	20.1	**— ^h^**	**39.6 ± 2.1**	**36.3 ± 2.0**	**23.4 ± 1.8**	**1.88 (1.49, 2.38)**	**2.47 (1.88, 3.24)**

SE: standard error; AOR: adjusted odds ratio; 95% CI: 95% confidence intervals. ^a^ Sweet food intake was measured using 4 questions and included chocolate/candy, doughnuts/sweet rolls/Danish/muffins/Pop-Tarts, cookies/cake/pie/brownies, and ice cream/frozen desserts. ^b^ χ^2^ tests were used for each variable to examine differences across categories. Variables with *p* < 0.05 were bolded. ^c^ All variables were included in one multinomial logistic regression model (unweighted *n* = 3937 without missing data). To increase sample sizes, sweet food intake of 0 times/day was combined with >0 to <1 time/day. The reference category was consuming sweet foods < 1 time/day. Significant findings are bolded based on the 95% confidence intervals, which does not include 1. ^d^ Weighted percent may not add up to 100% because of rounding. ^e^ Unweighted sample size. ^f^ Based on the following question: “Thinking about the past year, during the COVID-19 pandemic, how often did you rely on only a few kinds of low-cost food to feed yourself or household members because there wasn’t enough money to buy food?”. ^g^ Based on calculated body mass index (BMI) (kg/m^2^): underweight/healthy weight, BMI < 25; overweight, BMI 25 to <30; obesity, BMI ≥ 30. ^h^ Data were suppressed due to a small sample size.

**Table 2:** Associations between characteristics of respondents (US adults) and consuming sugar-sweetened beverages (SSB) during the COVID-19 pandemic, SummerStyles Survey, 2021.

	All Respondents	SSB ^a^ Intake during the Past Month					
		Bivariate Analysis ^b^				Multinomial Analysis ^c^	
Characteristic	% ^d^	0 Times/Day % ^d^ ± SE	>0 to <1 Time/Day % ^d^ ± SE	1 to <2 Times/Day % ^d^ ± SE	≥2 Times/Day % ^d^ ± SE	1 to <2 Times/Day AOR (95% CI)	≥2 Times/Day AOR (95% CI)
Total (*n* = 4034) ^e^	100	14.9 ± 0.6	30.5 ± 0.9	24.7 ± 0.8	29.9 ± 0.9	—	—
Age							
18–24 years	10.6	**12.6 ± 2.8**	**40.0 ± 4.2**	**25.2 ± 3.7**	**22.2 ± 3.6**	0.69 (0.43, 1.12)	**0.47 (0.28, 0.79)**
25–44 years	34.8	**12.4 ± 1.1**	**32.3 ± 1.6**	**24.2 ± 1.4**	**31.1 ± 1.6**	0.81 (0.61, 1.07)	0.80 (0.61, 1.05)
45–64 years	33.5	**13.9 ± 1.0**	**29.2 ± 1.3**	**24.7 ± 1.2**	**32.2 ± 1.3**	0.96 (0.76, 1.22)	1.04 (0.83, 1.30)
≥65 years	21.1	**21.7 ± 1.3**	**24.6 ± 1.4**	**25.3 ± 1.4**	**28.4 ± 1.5**	Reference	Reference
Sex							
Male	48.6	**14.1 ± 0.9**	**29.5 ± 1.2**	**23.5 ± 1.1**	**33.0 ± 1.2**	1.07 (0.88, 1.30)	**1.51 (1.25, 1.81)**
Female	51.4	**15.7 ± 0.9**	**31.4 ± 1.3**	**25.8 ± 1.2**	**27.1 ± 1.2**	Reference	Reference
Race/ethnicity							
Black, non-Hispanic	11.7	**14.5 ± 2.5**	**35.1 ± 3.1**	**24.8 ± 2.7**	**25.6 ± 2.8**	0.90 (0.65, 1.26)	**0.64 (0.45, 0.91)**
Hispanic	16.3	**9.0 ± 1.4**	**34.2 ± 2.8**	**26.1 ± 2.5**	**30.7 ± 2.7**	1.11 (0.81, 1.52)	0.88 (0.65, 1.19)
Other/multiracial, non-Hispanic	8.8	**14.0 ± 2.2**	**32.6 ± 3.1**	**25.3 ± 2.9**	**28.0 ± 3.0**	1.05 (0.73, 1.52)	1.04 (0.74, 1.46)
White, non-Hispanic	63.2	**16.6 ± 0.8**	**28.3 ± 1.0**	**24.2 ± 0.9**	**30.8 ± 1.0**	Reference	Reference
Education level							
High school or less	38.4	**12.9 ± 1.1**	**27.1 ± 1.5**	**22.6 ± 1.4**	**37.4 ± 1.6**	1.13 (0.86, 1.48)	**1.98 (1.55, 2.53)**
Some college	30.0	**15.1 ± 1.1**	**30.9 ± 1.6**	**26.5 ± 1.5**	**27.4 ± 1.5**	1.10 (0.87, 1.39)	**1.33 (1.06, 1.68)**
College graduate	31.6	**17.2 ± 1.1**	**34.1 ± 1.4**	**25.4 ± 1.3**	**23.3 ± 1.2**	Reference	Reference
Marital status							
Married	57.1	14.9 ± 0.8	30.4 ± 1.0	25.0 ± 1.0	29.7 ± 1.0	Reference	Reference
Not married	42.9	15.0 ± 1.1	30.5 ± 1.5	24.3 ± 1.4	30.2 ± 1.5	1.14 (0.91, 1.42)	1.14 (0.92, 1.41)
Annual household income							
<$35,000	19.9	**16.1 ± 1.8**	**24.8 ± 2.1**	**19.5 ± 1.9**	**39.6 ± 2.4**	0.82 (0.57, 1.17)	1.35 (0.99, 1.85)
$35,000–$74,999	27.5	**11.7 ± 1.1**	**31.1 ± 1.8**	**27.7 ± 1.7**	**29.4 ± 1.6**	1.16 (0.90, 1.50)	1.16 (0.90, 1.48)
$75,000–$99,999	13.7	**12.5 ± 1.5**	**31.7 ± 2.3**	**24.6 ± 2.1**	**31.2 ± 2.3**	1.03 (0.77, 1.38)	1.22 (0.93, 1.60)
≥$100,000	38.9	**17.5 ± 1.0**	**32.4 ± 1.3**	**25.2 ± 1.2**	**24.9 ± 1.2**	Reference	Reference
Currently have children (<18 years) (*n* = 4032)							
Yes	27.6	**11.0 ± 1.0**	**28.4 ± 1.6**	**25.8 ± 1.5**	**34.8 ± 1.7**	**1.28 (1.02, 1.62)**	**1.65 (1.31, 2.08)**
No	72.4	**16.4 ± 0.8**	**31.3 ± 1.1**	**24.2 ± 1.0**	**28.1 ± 1.0**	Reference	Reference
Food insecurity ^f^ (*n* = 4026)							
Often/sometimes	21.9	**9.2 ± 1.3**	**29.5 ± 2.1**	**26.7 ± 2.0**	**34.5 ± 2.0**	**1.35 (1.02, 1.79)**	1.26 (0.97, 1.65)
Seldom	12.8	**10.5 ± 1.6**	**31.2 ± 2.5**	**27.1 ± 2.4**	**31.2 ± 2.5**	1.28 (0.95, 1.71)	1.28 (0.96, 1.70)
Never	58.8	**17.7 ± 0.8**	**30.6 ± 1.1**	**24.3 ± 1.0**	**27.4 ± 1.0**	Reference	Reference
Do not know or not sure	6.5	**18.2 ± 3.4**	**29.7 ± 4.0**	**16.7 ± 3.1**	**35.3 ± 4.2**	0.74 (0.44, 1.25)	1.20 (0.77, 1.87)
Weight status ^g^ (*n* = 3957)							
Underweight/healthy weight	33.1	16.3 ± 1.2	32.8 ± 1.6	24.0 ± 1.5	26.9 ± 1.5	Reference	Reference
Overweight	32.3	14.2 ± 1.1	29.1 ± 1.5	24.8 ± 1.4	32.0 ± 1.5	1.16 (0.91, 1.47)	1.19 (0.95, 1.50)
Obesity	34.6	14.3 ± 1.1	29.2 ± 1.5	25.5 ± 1.4	31.0 ± 1.5	1.10 (0.87, 1.41)	1.00 (0.79, 1.27)
Metropolitan status							
Nonmetropolitan	13.4	**11.6 ± 1.7**	**25.9 ± 2.3**	**26.1 ± 2.2**	**36.4 ± 2.4**	1.34 (1.00, 1.81)	**1.34 (1.02, 1.76)**
Metropolitan	86.6	**15.4 ± 0.7**	**31.2 ± 1.0**	**24.5 ± 0.9**	**28.9 ± 0.9**	Reference	Reference
Census regions of residence							
Northeast	17.3	**18.7 ± 1.7**	**26.3 ± 1.9**	**21.9 ± 1.8**	**33.2 ± 2.0**	1.02 (0.77, 1.35)	1.07 (0.83, 1.37)
Midwest	20.8	**13.8 ± 1.3**	**30.8 ± 1.8**	**28.0 ± 1.8**	**27.4 ± 1.8**	1.25 (0.97, 1.61)	0.82 (0.64, 1.06)
South	38.1	**15.0 ± 1.1**	**30.2 ± 1.5**	**22.3 ± 1.3**	**32.5 ± 1.5**	Reference	Reference
West	23.8	**13.1 ± 1.2**	**33.6 ± 1.9**	**27.7 ± 1.8**	**25.6 ± 1.7**	1.18 (0.92, 1.52)	0.79 (0.61, 1.02)
Changes in SSB intake since start of the COVID-19 pandemic (*n* = 4004)							
Less than usual	20.7	**18.3 ± 1.7**	**36.3 ± 2.1**	**22.4 ± 1.8**	**23.1 ± 1.7**	**0.72 (0.56, 0.92)**	**0.60 (0.47, 0.77)**
Same as usual	68.8	**15.8 ± 0.8**	**28.9 ± 1.0**	**25.0 ± 1.0**	**30.2 ± 1.0**	Reference	Reference
More than usual	10.6	**— ^h^**	**29.5 ± 3.2**	**26.4 ± 2.9**	**43.0 ± 3.3**	**1.54 (1.05, 2.26)**	**2.23 (1.58, 3.16)**

SSBs: sugar-sweetened beverages; SE: standard error; AOR: adjusted odds ratio; 95% CI: 95% confidence intervals. ^a^ SSB intake was measured using 5 questions and included regular sodas, sweetened coffee/tea drinks fruit drinks (excluding 100% juice), sports drinks, and energy drinks. ^b^ χ^2^ tests were used for each variable to examine differences across categories. Variables with *p* < 0.05 were bolded. ^c^ All variables were included in one multinomial logistic regression model (unweighted *n* = 3922 without missing data). To increase sample sizes, SSB intake of 0 times/day was combined with >0 to <1 time/day. The reference category was consuming SSB <1 time/day. Significant findings are bolded based on the 95% confidence intervals, which does not include 1. ^d^ Weighted percent may not add up to 100% because of rounding. ^e^ Unweighted sample size. ^f^ Based on the following question: “Thinking about the past year, during the COVID-19 pandemic, how often did you rely on only a few kinds of low-cost food to feed yourself or household members because there wasn’t enough money to buy food?”. ^g^ Based on calculated body mass index (BMI) (kg/m^2^): underweight/healthy weight, BMI < 25; overweight, BMI 25 to <30; obesity, BMI ≥ 30. ^h^ Data were suppressed due to a small sample size.

## Data Availability

Data sharing is not applicable to this article.

## Supplementary Materials

The following supporting information can be downloaded at: https://www.mdpi.com/article/10.3390/nu15102363/s1.
