# Supplementary material for: Characteristics Associated with Being a High Consumer of Sweet Foods and Sugar-Sweetened Beverages among US Adults during the COVID-19 Pandemic, 2021
Source: Nutrients. 2023 May 18;15(10):2363. doi: 10.3390/nu15102363 (PMC10222205; doi:10.3390/nu15102363)
Supplement: Supplementary file 1 [file nutrients-15-02363-s001.zip › nutrients-2380520-supplementary.pdf]

nutrients MDPI

Supplemental Table S1. Associations between sociodemographic characteristics of respondents (US adults) and consuming sweet food and SSB during COVID-19 pandemic when the variable on changes in sweet food and SSB intake since the start of the COVID-19 pandemic was removed from the model, SummerStyles Survey, 2021 (n = 3948).

	Sweet Food Intake during the Past Month		SSB Intake during the Past Month	
	Multinomial Analysis [a]		Multinomial Analysis [b]	
**Characteristic**	**1 to <2 Times/Day AOR (95% CI)**	**≥2 Times/Day AOR (95% CI)**	**1 to <2 Times/Day AOR (95% CI)**	**≥2 Times/Day AOR (95% CI)**
**Age**				
18–24 years	0.83 (0.53, 1.29)	**0.50 (0.27, 0.91)**	0.77 (0.48, 1.23)	**0.55 (0.33, 0.91)**
25–44 years	**0.65 (0.51, 0.84)**	**0.71 (0.51, 0.97)**	0.83 (0.63, 1.10)	0.87 (0.67, 1.14)
45–64 years	**0.68 (0.55, 0.84)**	**0.64 (0.49, 0.83)**	0.97 (0.76, 1.22)	1.08 (0.86, 1.34)
≥65 years	Reference	Reference	Reference	Reference
**Sex**				
Male	0.84 (0.70, 1.00)	0.86 (0.69, 1.07)	1.04 (0.88, 1.30)	**1.45 (1.20, 1.74)**
Female	Reference	Reference	Reference	Reference
**Race/ethnicity**				
Black, non-Hispanic	**0.63 (0.45, 0.86)**	**0.62 (0.40, 0.96)**	0.93 (0.66, 1.30)	**0.64 (0.45, 0.90)**
Hispanic	0.85 (0.62, 1.15)	1.00 (0.70, 1.45)	1.13 (0.83, 1.54)	0.91 (0.67, 1.23)
Other/multiracial, non-Hispanic	0.88 (0.64, 1.21)	0.70 (0.44, 1.10)	1.07 (0.74, 1.53)	1.06 (0.75, 1.49)
White, non-Hispanic	Reference	Reference	Reference	Reference
**Education level**				
High school or less	0.85 (0.67, 1.11)	0.92 (0.69, 1.23)	1.12 (0.86, 1.47)	**1.95 (1.54, 2.48)**
Some college	0.98 (0.79, 1.22)	0.87 (0.66, 1.15)	1.13 (0.89, 1.42)	**1.36 (1.08, 1.70)**
College graduate	Reference	Reference	Reference	Reference
**Marital status**				
Married	Reference	Reference	Reference	Reference
Not married	0.93 (0.75, 1.14)	1.04 (0.81, 1.33)	1.13 (0.90, 1.41)	1.15 (0.93, 1.42)
**Annual household income**				
<$35,000	0.93 (0.67, 1.28)	1.34 (0.93, 1.93)	0.78 (0.55, 1.12)	1.33 (0.97, 1.81)
$35,000–$74,999	0.99 (0.78, 1.26)	0.96 (0.72, 1.29)	1.14 (0.89, 1.47)	1.15 (0.90, 1.47)
$75,000–$99,999	1.15 (0.89, 1.49)	0.94 (0.66, 1.33)	1.02 (0.77, 1.36)	1.25 (0.93, 1.63)
≥$100,000	Reference	Reference	Reference	Reference
**Currently have children (<18 years)**				
Yes	1.12 (0.90, 1.40)	0.90 (0.67, 1.21)	**1.32 (1.04, 1.67)**	**1.66 (1.32, 2.09)**
No	Reference	Reference	Reference	Reference
**Food insecurity**				
Often/sometimes	**1.30 (1.004, 1.67)**	**1.49 (1.09, 2.03)**	**1.38 (1.05, 1.81)**	**1.34 (1.03, 1.74)**
Seldom	1.18 (0.91, 1.54)	1.31 (0.93, 1.83)	1.28 (0.96, 1.72)	1.25 (0.94, 1.66)
Never	Reference	Reference	Reference	Reference
Do not know or not sure	1.04 (0.66, 1.64)	0.74 (0.41, 1.33)	0.72 (0.43, 1.21)	1.16 (0.74, 1.81)
**Weight status**				
Underweight/healthy weight	Reference	Reference	Reference	Reference
Overweight	1.04 (0.84, 1.30)	0.99 (0.75, 1.32)	1.14 (0.89, 1.44)	1.18 (0.94, 1.48)
Obesity	0.93 (0.73, 1.17)	1.00 (0.76, 1.32)	1.10 (0.87, 1.40)	1.02 (0.81, 1.30)
**Metropolitan status**				
Nonmetropolitan	**0.65 (0.50, 0.86)**	1.05 (0.76, 1.45)	**1.38 (1.03, 1.84)**	**1.33 (1.01, 1.73)**
Metropolitan	Reference	Reference	Reference	Reference
**Census regions of residence**				
Northeast	1.10 (0.85, 1.41)	1.27 (0.92, 1.74)	1.04 (0.79, 1.38)	1.10 (0.88, 1.41)
Midwest	**1.40 (1.11, 1.76)**	1.11 (0.82, 1.51)	**1.30 (1.01, 1.67)**	0.84 (0.65, 1.08)
South	Reference	Reference	Reference	Reference
West	0.99 (0.78, 1.26)	0.99 (0.72, 1.34)	1.20 (0.93, 1.54)	0.78 (0.61, 1.01)

SSBs: sugar-sweetened beverages; AOR: adjusted odds ratio; 95% CI: 95% confidence intervals. [a] All variables were included in one multinomial logistic regression model. To increase sample sizes, sweet food intake of 0 times/day was combined with >0 to <1 time/day. The reference category was consuming sweet foods < 1 time/day. Significant findings are bolded based on the 95% confidence intervals, which does not include 1. [b] All variables were included in one multinomial logistic regression model. To increase sample sizes, SSB intake of 0 times/day was combined with >0 to <1 time/day. The reference category was consuming SSB < 1 time/day. Significant findings are bolded based on the 95% confidence intervals, which does not include 1.
